# Chemical Composition, Functional and Antioxidant Properties of Dietary Fibre Extracted from Lemon Peel after Enzymatic Treatment

**DOI:** 10.3390/molecules29010269

**Published:** 2024-01-04

**Authors:** Vanesa Núñez-Gómez, Marta San Mateo, Rocío González-Barrio, Mª Jesús Periago

**Affiliations:** Department of Food Technology, Food Science and Nutrition, Faculty of Veterinary Sciences, Regional Campus of International Excellence “Campus Mare Nostrum”, Biomedical Research Institute of Murcia (IMIB-Arrixaca-UMU), University of Murcia, 30100 Murcia, Spain; marta.sanm@um.es (M.S.M.); rgbarrio@um.es (R.G.-B.); mjperi@um.es (M.J.P.)

**Keywords:** soluble dietary fibre, (poly)phenols, by-product, swelling capacity, water retention capacity, functional ingredient, valorisation

## Abstract

Lemon peel represents an interesting by-product owing to its content of dietary fibre (DF) and (poly)phenols, which is of great importance for its valorisation. Hence, the objective of this study was to characterise the DF, total phenolic content (TPC), and antioxidant capacity of two lemon-peel-derived ingredients using two different methods (drying with warm air and enzymatic hydrolysis with pectinesterase). The analysis included a DF assessment, followed by neutral sugars characterisation through GC-FID and uronic acids determination via colorimetry. Subsequently, TPC and antioxidant capacity using the FRAP method were quantified through spectrophotometry. The swelling capacity (SWC), water retention capacity (WRC), and fat absorption capacity (FAC) were also determined as functional properties. It was observed that pectinesterase treatment led to a reduction in soluble DF and an increase in insoluble DF. This treatment also affected the pectin structure, thereby diminishing its ability to absorb water and fat within its matrix. The TPC was also reduced, resulting in a decrease in antioxidant capacity. Conversely, employing warm air exhibited a noteworthy increase in antioxidant capacity. This underscores its crucial contribution to the valorisation of lemon peel, not only by diminishing the environmental impact but also by enabling the acquisition of fibre ingredients with a noteworthy antioxidant capacity.

## 1. Introduction

The growing generation of by-products in the agro-industry has heightened the necessity for developing techniques that enable their reintegration into the food chain [1]. This is crucial for implementing the increasingly prevalent circular economy models [2]. Notably, lemons, being a globally consumed fruit, contain components with health-enhancing properties, including dietary fibre, vitamins, minerals, and bioactive compounds, such as (poly)phenols and carotenoids [3].

Concerning by-products from lemons, they typically originate from the peel or pulp [1]. This is largely due to the predominant use of the fruit for juice production [4]. Citrus peel, in general, is rich in dietary fibre, flavonoids, carotenoids, and essential oils [5]. Specifically, the albedo, constituting the inner part and being the primary component of the peel, serves as the main source of fibre in the fruit. It is considered high-quality fibre owing to its association with bioactive compounds integrated into its matrix [6]. When treating these by-products, the extraction conditions are crucial. This includes solvents, enzymes, or physical conditions, all of which significantly influence the characteristics and the composition of the ingredients obtained from these by-products [7,8,9]. Furthermore, depending on the method selected, there are several advantages and disadvantages. In this sense, enzymatic methods have a higher yield and allow for obtaining fibre fractions with higher purity. On the other hand, these methods require more laborious protocols, which need a greater number of reagents and are therefore more expensive. Conventional drying processes, in turn, are cheaper and do not require reagents, thus reducing the use of water and energy, but, in contrast, the fibres obtained are of lower purity [10].

Dietary fibre has several health effects, such as intestinal motility, an effect on postprandial glucose and insulin response, a cholesterol-lowering effect, and a prebiotic effect, among others [11]. In addition, dietary fibre may have an antioxidant effect, as it may have (poly)phenols attached through hydrogen bonds to the polysaccharides, which are known as non-extractable (poly)phenols (NEPPs) [12,13]. Extractable (poly)phenols (EPP), on the other hand, are those that are not bound to the fibre matrix and can therefore be extracted with organic solvents, while NEPPs must be extracted by acid or alkaline hydrolysis. As for EPPs, they can be absorbed in the first parts of the gastrointestinal tract because of their free form. NEPPs, on the other hand, must be released from the fibre, and this release occurs to a greater extent in the colon after fermentation of the dietary fibre [14,15].

Therefore, dietary fibre bound to (poly)phenols might have an antioxidant effect associated with the presence of these compounds. Upon ingestion, these (poly)phenols facilitate the neutralisation of free radicals by donating additional electrons. In this process, they prevent cell damage and serve as a protective barrier against chronic diseases associated with oxidative stress, including heart disease, diabetes, and specific types of cancer [16].

The objective of this study was to analyse the composition of dietary fibre and the (poly)phenol content and antioxidant capacity of two different samples, which were obtained from lemon peel by-products dried using hot air or treated through enzymatic hydrolysis with pectinesterase, in order to evaluate their nutritional value and their functional properties as fibre-rich food ingredients.

## 2. Results and Discussion

### 2.1. Dietary Fibre Content

**Figure 1:**
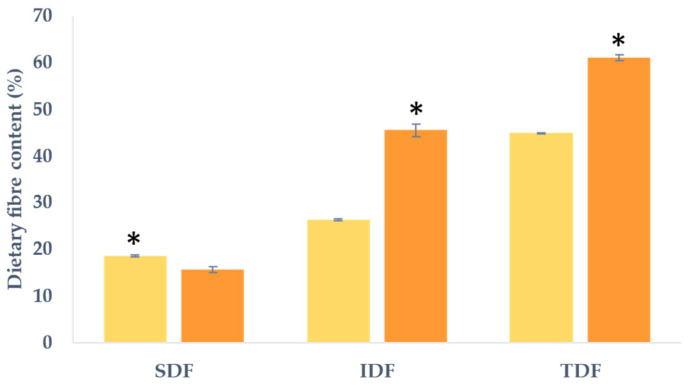
Dietary fibre composition expressed as percentage g/100 g of dry weight (d.w.) for lemon peel (■ LP) and lemon peel hydrolysed by pectinesterase (■ LPp). SDF (soluble dietary fibre); IDF (insoluble dietary fibre); TDF (total dietary fibre). Values are expressed as mean ± SD (*n* = 3). * Indicates significant differences (*p* < 0.05) among the samples for each fibre fraction (SDF, IDF, and TDF).

Figure 1 illustrates the percentages of total dietary fibre (TDF), soluble dietary fibre (SDF), and insoluble dietary fibre (IDF) expressed in g/100 g, revealing statistically significant differences between the two samples of lemon peel (LP) and lemon peel treated with pectinesterase (LPp). The TDF content was 45% for the LP sample and 61% for the LPp, which was notably higher, indicating a significant reduction in fibre content in the sample that did not undergo pectinesterase hydrolysis or that the treatment with pectinesterases in the LPp sample results in the removal of other components that comprise the peel when the supernatant is removed after the enzymatic treatment, leading to fibre concentration. The TDF content observed for both samples closely aligns with that documented by the USDA for lemon peel, which is 58% in dry weight (calculated based on the water content) [17].

In terms of the SDF and IDF content, significant disparities were noted between the two samples. In both instances, the IDF fraction prevailed over SDF. When assessed as a percentage of the total fibre, the LP sample exhibited a higher proportion of SDF (41.5%) compared to the LPp sample (25.5%). Conversely, LPp displayed a greater percentage of IDF at 74.5% as opposed to LP, which registered 58.5%. The variance in SDF proportion can be attributed to the enzymatic treatment with pectinesterases to which the LPp sample has been subjected. These enzymes hydrolysed the polysaccharide primarily contributing to this fibre fraction, pectin, thus yielding smaller polymers exhibiting characteristics reminiscent of SDF.

A literature review estimated the amount of fibre in dried citrus peel to be 57%, with the results obtained in our study falling near this value [18]. Other authors estimated slightly higher average values. Czech et al. [19] described a TDF amount of 64%, while Rafiq et al. [6] showed values in a range between 60% and 68%. Another study reported similarity with the LPp sample in terms of the total percentage and different fractions; these authors described a TDF content for peels from different citrus fruits between 62% and 64%, of which the SDF was between 13% and 14% and the IDF was between 49% and 50% [20]. Moreover, when comparing these results with those obtained by other authors for soy fibre extracted with an enzymatic cellulase treatment, the results were also similar, reporting a mean content of 63% of IDF and 6.5% of SDF [21]. This outcome suggests that citrus-fruit-peel-derived products could be of interest to the food industry given their potential uses as functional components in confectionery, bakery items, or the production of high-fibre foods.

### 2.2. Dietary Fibre Characterisation by GC-FID

Table 1 presents the neutral sugars and uronic acid profiles of both lemon peel samples expressed as percentages. Moreover, Figure S1 shows the chromatograms obtained for the neutral sugars in both samples and in the standard mix. Notable differences in the content of all analysed sugars and uronic acids were observed between samples. In the LP sample, the primary sugars were arabinose, followed by galactose, while glucose, xylose, and mannose exhibited percentages ranging from 7% to 9%. Rhamnose and fucose were present in lower concentrations. In contrast, the LPp sample featured galactose, rhamnose, and glucose as the major sugars, with intermediate percentages of arabinose and mannose falling within the 4% to 9% range, and lower proportions of fucose and xylose. These findings in the LP sample align with those of previous researchers who reported that dried citrus peel samples predominantly contain arabinose, galactose, and glucose, underscoring the direct impact of the treatment on the proportion of neutral sugars [20]. The uronic acid content in LP was higher than the LPp due to the fact that LP was not treated with pectinesterase. However, the values for both samples were between those previously reported by other authors (12–24%) [9,20,22]. When comparing the results for both fractions with those obtained by other authors for a broccoli by-product fibre extracted with an anzymatic method and another as a control, the results agree with those obtained in our study, showing that the arabinose, xylose, and mannose content were higher in the control samples, as is observed for these neutral sugars in the LP sample [23].

The proportions of the primary dietary fibre polysaccharides, namely cellulose, hemicellulose, and pectin, were calculated from the neutral sugars, as shown in Table 1. In both samples, pectin emerged as the dominant polysaccharide, constituting approximately 73% of the composition, with no significant differences observed between the two samples. This outcome may be attributed to the pectin percentage calculation, which considers monosaccharides. In the case of the hydrolysed peel, these monosaccharides exist in a free state rather than forming more complex structures, such as pectin. However, notable variations were detected in the percentages of cellulose and hemicellulose. In the LP sample, hemicellulose prevailed at 18.7%, overcoming cellulose at 8.2%. Conversely, in the LPp sample, cellulose was the dominant component, accounting for 18.7%, while hemicellulose constituted 7.5% of the total polysaccharide content. These results were in agreement with those showed for the dietary fibre composition (Table 1), where it can be seen that LPp sample had more insoluble fibre, mainly due to the cellulose content. In accordance with findings from other authors, citrus residues generally display a composition expressed as a percentage, with approximately 43% pectin, 11% hemicellulose, and 9% cellulose, values that closely resemble those obtained in our samples [18].

When calculating other parameters related to the fibre composition (Table 2), significant differences are observed in the mannose-to-xylose ratio, which reflects the proportion of mannose relative to xylose in hemicellulose. Previous studies, such as the investigation by Peng et al., have reported that water-soluble hemicelluloses from maize stems exhibit a higher mannose content and a lower xylose content [24]. This could elucidate why the hemicellulose in LPp, with a greater mannose contribution, was primarily composed of water-soluble hemicelluloses. Conversely, in the case of LP, which exhibited the lowest mannose contribution and thus the highest xylose content, non-water-soluble hemicelluloses were the predominant constituents.

Moreover, additional indices, such as pectin linearity, the contribution of rhamnose and uronic acids to pectins, and RG-I branching, were computed, and significant differences were also observed for them. The results indicated that pectin linearity was similar, with no significant differences observed. This parameter holds significance as it determines the ability to create emulsions, which is favoured when linearity is higher due to the enhanced hydration properties of pectin [25]. Nevertheless, it is expected that the pectin chains may differ between the samples due to the enzymatic action.

Additionally, the ratio of rhamnose to uronic acid was significantly higher in LPp compared to LP, indicating the presence of longer RG-I domains. The RG-I domains represent around 7–14% of the whole pectin and are composed of rhamnose and galacturonic acids. These are the ones that give the branched structure, and they are crucial as they confer elasticity and viscosity among other gelling properties. Concerning RG-I branching, which reflects the extent of branching in the pectin molecules, LP exhibited the highest values. These findings align with the research of other authors, who have demonstrated that variations in the extraction process can directly influence the structure of the obtained pectin [26].

The composition of LPp suggests higher solubility, as indicated by the mannose-to-xylose ratio. Conversely, in terms of the contribution of rhamnose and uronic acids to pectin, LPp exhibited higher values, which means longer branches. This signifies that pectinesterase treatment led to the hydrolysis of pectin, thereby releasing uronic acids, maintaining rhamnose, and increasing this ratio. The RG-I branching index, which signifies the presence of galactose and arabinose side chains attached to the rhamnose of the pectin, shows more chain domains. To sum up, LP had more branches, but they are shorter, and LPp had less branches but longer ones. As such, LPp molecules were more flexible, which allowed them to interact less with each other, resulting in less rigid structures that allow, for example, a better stabilisation of emulsions [25,27]. Another parameter to characterise the pectin structure in the isolated samples was the length of the side chains, which are mainly formed by arabinose and galactose units. The length of the side chains was highest in LP, indicating that pectin from this sample may have stronger molecular interactions, thus leading to more consistent structures and making this characteristic interesting at an industrial level [27]. This outcome is due to LP preserving the integrity of lemon pectin, as it has not undergone pectinesterase treatment, thereby retaining the branching structure.

### 2.3. Functional Properties

Swelling capacity, water retention capacity, and fat absorption capacity were measured to evaluate the functional properties of LP and LPp (Table 3). There were significant differences in all of the functional properties determined, with the LP sample showing higher mean values compared to the LPp sample. The LP sample exhibits better hydration properties and contains a higher amount of SDF, allowing it to retain more water and fat within its matrix. These properties suggest its potential use as a natural added ingredient in bakery products and to fortify dairy foods and develop low-fat products [28,29]. These results are supported by those reported by Rivas et al. (2022), who reported that enzymatic treatment reduces hydration properties of dietary fibre isolated from broccoli by-products [23]. In a study by Huang et al., untreated citrus peel displayed mean functional property values of SWC at 8 mL/g, WRC at 8 g/g, and FAC at 2 g/g. The hydration property values were similar to those of the LP sample but differed from the data obtained for the treated sample (LPp), except for FAC [5].

On the other hand, according to Zhang et al., SDF from lemon peel exhibited higher values for all three functional properties compared to the data obtained in this study for LPp [30]. These results indicate that after treatment with pectinesterase, the hydration and fat absorption properties decrease primarily due to the changes in the pectin structure discussed in the previous section.

**Figure 2:**
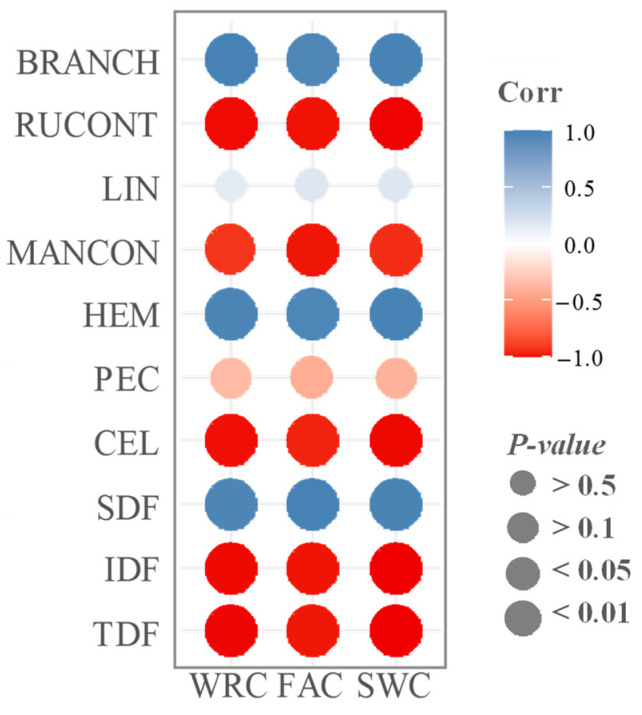
Correlation analysis between functional properties (FAC (fat absorption capacity), SWC (swelling capacity), and WRC (water retention capacity)) and fibre composition (TDF (total dietary fibre), IDF (insoluble dietary fibre), SDF (soluble dietary fibre), PEC (pectin content), CEL (cellulose content), HEM (hemicellulose content), MANCON (mannose contribution to hemicellulose), RUCONT (rhamnose–uronic acid contribution to pectin), LIN (linearity of pectin), and BRANCH (branching of pectin)) in the lemon peel fibre samples.

A correlation analysis, illustrated in Figure 2, was carried out to explore the relationship between the functional properties, dietary fibre composition, and characterisation. The results indicated a positive correlation between the three analysed properties and SDF, whereas a negative correlation was observed with the presence of TDF and IDF. Regarding the correlation of functional properties with the mannose/xylose contribution index, all three properties exhibited a negative correlation. This suggests that in the analysed samples, a higher presence of soluble hemicellulose does not enhance functional properties, highlighting its association with the pectin structure. Notably, the correlation with the mannose/xylose contribution index revealed a negative impact on the functional properties, particularly with the contribution of rhamnose and uronic acids. As described earlier, longer chain lengths result in increased stiffness, thus hindering the formation of compact structures that can retain water or oils [27].

Contrary to previous findings by Belkheiri et al. [26], greater RG-I branching in the samples was linked to improved functional properties. It is essential to note that the individual characteristics of pectin are less crucial than the overall structure of pectin in determining its flexibility. In this instance, LPp molecules exhibited higher flexibility, indicating a lack of rigid structures and, consequently, reduced capacity to retain water and oil within its composition.

### 2.4. Total Phenolic Content

**Figure 3:**
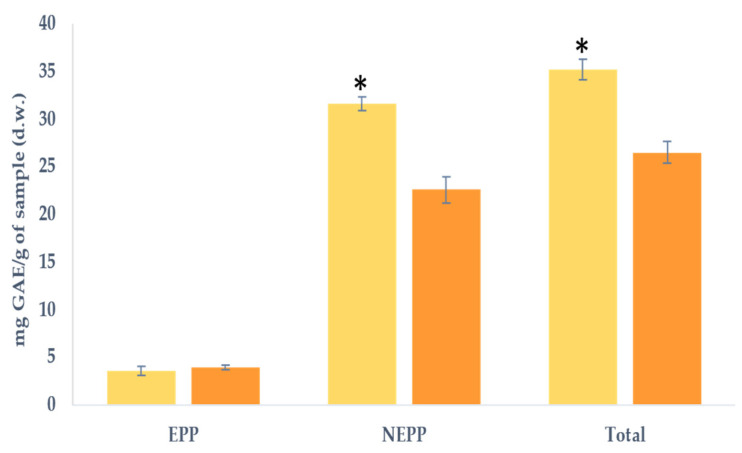
Total phenolic content (TPC) expressed as mg of gallic acid equivalents (GAE)/g of the sample in dry weight (d.w.) for lemon peel (■ LP) and lemon peel hydrolysed by pectinesterase (■ LPp). EPP (extractable (poly)phenols); NEPP (non-extractable (poly)phenols). Values are expressed as mean ± SD (*n* = 3). * Indicates significant differences (*p* < 0.05) among the samples.

Figure 3 displays the TPC in the EPP and NEPP fractions of both samples. It is noteworthy that there were no significant differences in EPP between the samples, with a mean value of 3.7 mg GAE/g. Although an increase in EPP could be expected due to the potential enzymatic release, it should be noted that these compounds could be removed after the application of ethanol to precipitate the soluble polysaccharides. Regarding the NEPP, a notable observation is that the LP fraction exhibited the highest significant content, being 1.4 times greater than that observed for LPp. The lower presence in the LPp may be due to the aforementioned effect, which resulted in an enzymatic release of compounds.

Singh et al. (2020) reported a range of TPC in lemon peel from 88 mg GAE/g to 190 mg GAE/g, with values akin to those found in our sample (LP, 150.4 mg GAE/g) [3]. Other studies have cited TPC content between 65 and 72 mg GAE/g, aligning closely with our findings [7,8]. Treatment with pectinesterase resulted in reduced NEPP content, leading to a lower TPC in the LPp sample. This suggests that the applied processing may trigger the release or loss of (poly)phenols from the fibre matrix.

Although no individual (poly)phenols have been identified in this study, it should be noted that the major (poly)phenols described by other authors are eriocitrin, hesperidin, rutin, and other compounds, such as limonin, that belong to the furanolactones group [31,32]. Moreover, the literature indicates variations in (poly)phenol content based on the extraction process [33]. In this case, for LPp, where ethanol was used to precipitate soluble polysaccharides, it may also contribute to removing part of the (poly)phenols and therefore lead to a lower content compared to LP, where no ethanol was used. In addition, the drying process may also affect the (poly)phenol content, as air drying is the least preservative for these compounds, although it should be noted that in our study, where this method was used, the total values are similar to those described by other authors, as previously indicated [33].

### 2.5. Antioxidant Capacity

**Figure 4:**
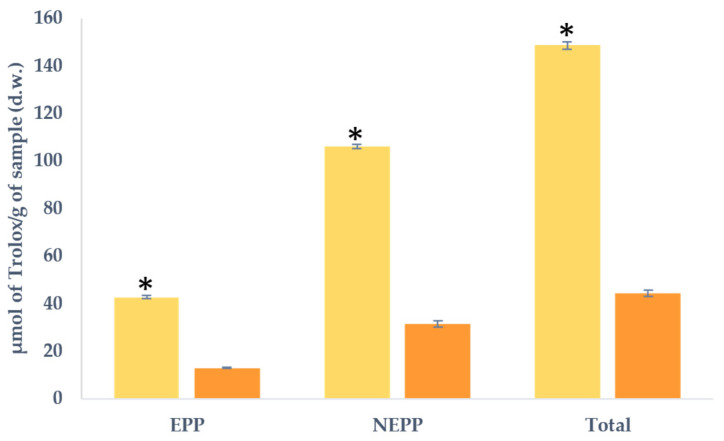
Antioxidant capacity expressed as µmol of Trolox/g of sample in dry weight (d.w.) for lemon peel (■ LP) and lemon peel hydrolysed by pectinesterase (■ LPp). EPP (extractable (poly)phenols); NEPP (non-extractable (poly)phenols). Values are expressed as mean ± SD (*n* = 3). * Indicates significant differences (*p* < 0.05) among the samples.

Figure 4 illustrates the antioxidant capacity of both samples, as assessed through FRAP in the EPP and NEPP extracts. Notably, significant variations were noted, indicating that the antioxidant capacity was higher in LP compared to LPp for both extracts and, consequently, in the overall content. The disparity in content was remarkable, with values 3.3 times higher for EPP, 3.4 times higher for NEPP, and 3.4 times higher for the total content in LP compared with LPp. It is important to acknowledge that these findings are in alignment with those observed for TPC. However, it is worth noting that the differing ratios may be attributed to other compounds influencing antioxidant capacity, such as carotenoids, which were not measured in the current study [34]. Additionally, there was a positive correlation observed for NEPP and the total content concerning antioxidant capacity.

It is notable that the values observed in this study appear to be lower than those reported by other researchers for lemon peel samples, ranging between 133 and 380 µmol/g. This disparity could potentially be attributed to variations in sample acquisition methods, differences in the extraction process employed for analysis, or the utilisation of different lemon varieties in the respective studies [7,8].

While other authors have reported a decline in antioxidant capacity due to hot air drying, such an effect was not evident in our study. It is important to note that the observed outcomes may not solely be attributable to this treatment or the presence of other bioactive compounds. The formation of Maillard compounds should also be considered, as they arise from heat treatment to the sugars present in the sample [35]. Although these compounds were not measured in the present study, they may potentially have been formed. As previously mentioned, the LPp sample underwent treatment with pectinesterase and precipitation with ethanol, which may contribute to the removal of part of the sugars present in this sample, thus resulting in lower production of these compounds in case they were formed.

All of the results taken together show that lemon peel is a valuable source of dietary fibre that is predominantly soluble and primarily comprises pectin; it also contains IDF due to its cellulose and hemicellulose components. It boasts good functional properties, including hydration and fat absorption properties. Moreover, the presence of (poly)phenols contribute to its antioxidant capacity.

In conclusion, upon treatment with pectinesterases, there were noticeable changes in the composition due to pectin hydrolysis. This process resulted in a reduction in the SDF percentage, an increase in the IDF content, a higher proportion of cellulose, and a diminished hemicellulose content. The alteration in the pectin structure adversely affected hydration and fat absorption properties. Simultaneously, processing led to a reduction in the (poly)phenol content, consequently the diminishing antioxidant capacity.

For all that, the treatment applied compromises further uses of the obtained ingredients and is a crucial step in the valorisation of lemon by-products. Because the valorisation of lemon peel by-products emerges as a crucial avenue, this will enable the acquisition of novel ingredients rich in dietary fibre and (poly)phenols with a notable antioxidant capacity. This approach holds promise for mitigating the environmental impact associated with substantial by-product generation at the agro-industrial level.

## 3. Materials and Methods

### 3.1. Samples

In the current study, we employed two samples derived from by-products of the citrus industry, specifically lemon peels. One sample was obtained by drying lemon peels with warm air in a chamber at 60 °C until constant weight (LP) (≈48 h), while the other sample underwent a hydrolysis process using pectinesterases (LPp), and, after the enzymatic treatment, the sample was subsequently dried using the same conditions described above. Both samples were ground, and the powders obtained were stored under refrigeration until analyses were performed.

### 3.2. Enzymatic Treatment

The lemon peel sample was mixed with water (1/1, *v*/*v*), and then pectin was hydrolysed by adding 1% of commercial pectinesterase (EC 3.1.1.11.) (pectin/NaCl, *w*/*v*) (Sigma, St. Louis, MO, USA). Samples were introduced to a water bath at 30 °C for 30 min at pH 7.5. To inactivate the enzyme, samples were introduced to a bath at 90 °C during 2 min, and after that they were put in cold water until reaching room temperature. Absolute ethanol was added in a proportion of 1/4, *v*/*v* to precipitate the soluble dietary fibre polysaccharides. In the following step, the solid phase was separated through centrifugation, and it was dried in a chamber at 60 °C until constant weight (≈48 h).

### 3.3. Dietary Fibre Quantification

Total dietary fibre (TDF), insoluble dietary fibre (IDF), and soluble dietary fibre (SDF) were determined through the enzymatic–gravimetric method described by Prosky et al. [36]. In short, an enzymatic digestion was carried out by using the Total Dietary Fibre Assay Kit (Megazyme Ltd., Wicklow, Ireland).

For analysis, 0.5 g of each sample was weighed and diluted with 40 mL of MES-TRIS Buffer. After homogenisation, 50 µL of α-amylase enzyme was added to facilitate starch hydrolysis, which was placed for 30 min at 100 °C and 60 rpm in a thermostatic bath. Subsequently, 100 µL of protease was added and maintained at 60 °C under the same conditions. Once digestion was completed, the mixture was cooled to 20 °C, and the pH was adjusted between 4.1 and 4.8 before adding 5 mL of 0.561 N HCl. Next, 200 µL of amyloglucosidase was introduced, and the mixture was incubated for 30 min at 60 °C. To separate the two phases, the Fibertec System E 1023 (Foss, Högänas, Sweden) was used. An initial filtration was performed to isolate the IDF. The volume obtained from the filtrate of the previous step was combined with a 95% ethanol solution at 65 °C and allowed to stand for one hour. Following this, the filtration procedure was repeated, and the SDF residue was collected.

Once the residues were obtained, their weight was calculated by determining the ash and protein content. This involved using a muffle furnace for the incineration of the residues at 525 °C during 24 h. Additionally, the Kjeldahl method was used to quantify both the total nitrogen and the protein [37].

### 3.4. Dietary Fibre Characterisation through Gas Chromatography

To determine the composition of dietary fibre polysaccharides, a process similar to that previously described for SDF and IDF was carried out, but only the TDF fraction was obtained. After enzymatic digestion, 95% ethanol was added at 65 °C, followed by centrifugation at 4500× *g* for 10 min, and the protocol described by Englyst et al. was followed [38]. Briefly, the supernatant was removed, and the residue was treated with 5 mL of 12 M H_2_SO_4_. It was maintained in a 35 °C bath for 30 min, with homogenisation every 10 min. Finally, the mixture was heated in a bath for 1 h at 100 °C, with homogenisation every 10 min. Once the acid hydrolysis was complete, breaking down the polysaccharide chains, sugar derivatisation was performed on the hydrolysed samples. The analysis was performed using a Gas Chromatography (GC) 7890B (Agilent, Machelen, Belgium). A mixture of neutral sugars (rhamnose, fucose, arabinose, xylose, mannose, galactose, and glucose) was used as the standard, and allose (2595-97-3, Thermo Scientific, Madrid, Spain) served as the internal standard. The results were expressed as a percentage.

Uronic acids were determined through the colorimetry method described by Scott (1979) [39] using the hydrolysed residue obtained in the previous extraction. In short, the sample was diluted with 2 M H_2_SO_4_ and 300 µL of 3% boric acid and 2% sodium chloride were added, followed by 5 mL of H_2_SO_4_. The mixture was then heated in a 70 °C bath for 40 min. After cooling, 200 µL of dimethylphenol was added, and after 15 min, the absorbance was measured at 400 nm and 450 nm to remove hexose interference. For quantification, galacturonic acid was used, and the results were expressed as a percentage.

The pectin, hemicellulose, and cellulose percentages and the pectin structure in the samples were estimated based on the calculations proposed by Houben et al. and Umaña et al. [40,41].

### 3.5. Functional Properties

To determine the hydration properties (SWC and WRC) of the fibre and the fat absorption capacity, three different functional properties were analysed based on the protocols previously published by Navarro-González et al. [42].

The WRC was measured by weighting 1 g of the sample, which was mixed with 30 mL of MilliQ water and then homogenised. After standing for 24 h, it was centrifuged at 3000 rpm for 20 min. The supernatant was removed, and the dry residue was weighed and kept for 18 h at 110 °C. The WRC was calculated according to the gravimetric difference.

For the SWC, 0.1 g of the sample was weighed, and 10 mL of MilliQ water was added. The mixture was mixed and left at room temperature for 18 h, followed by measuring the volume occupied by the dry residue in the graduated tube.

For the FAC, 4 g of the sample was weighed, and 24 mL of sunflower oil was added. The mixture was homogenised at 5 min intervals for 30 min. Subsequently, the mixture was centrifuged for 25 min at 3000 rpm, and the supernatant was removed. The residue with the adsorbed oil was weighed, and the calculation was performed according to the gravimetric difference.

### 3.6. (Poly)phenol Extraction and Total Phenolic Content Analysis

To extract (poly)phenols, two different extraction procedures described by Arranz et al. [43] were employed to yield both extractable (poly)phenols (EPP) and non-extractable (poly)phenols (NEPP). In short, to determine the EPP, 0.25 g of the sample was taken and blended with a solution of methanol and water acidified with 1% HCl (50/50, *v*/*v*) to achieve a pH close to 2. The samples were homogenised in an orbital shaker and subsequently centrifuged (4500× *g*, 10 min, room temperature) to collect the supernatant. This process was then repeated using a solution of acetone/water (70/30 *v*/*v*), and the resulting supernatant was combined with the previous one.

The pellet derived from the preceding process was used for determining the NEPP. This pellet was mixed with 10 mL of methanol/water/formic acid (79/19/1, *v*/*v*/*v*), which underwent a 20 h incubation at 85 °C, followed by centrifugation (4500× *g*, 10 min, room temperature). The collected supernatant was isolated, and the resultant residue was washed with the extraction solvent and mixed with the one previously obtained.

The total phenolic content (TPC) was analysed for both the EPP and the NEPP using the Folin–Ciocalteu method described by Singleton and Rossi (1965) [44]. This method involves an oxidation/reduction and colorimetric reaction. For the colorimetric assay, both a Folin–Ciocalteau reagent and Na_2_CO_3_ were added until approximately pH 10, where a blue chromophore emerged due to the reduction of phosphomolybdic-phosphotungstic complexes, leading to tungsten and molybdenum oxides. After 1 h, absorbance was measured at 750 nm using a UV–visible spectrophotometer (Evolution 300, Thermo-Scientific, Oxford, UK). Gallic acid (Riedelde Haën, Hannover, Germany) was used as the standard, and the TPC in the samples was expressed as mg of gallic acid equivalents (GAE)/g of the sample expressed in dry weight (d.w.).

### 3.7. Antioxidant Capacity Analysis

The antioxidant capacity analysis was determined in both fractions, EPP and NEPP, extracted following the protocol described above. For that, the ferric reducing antioxidant power (FRAP) assay was used following the protocol described by Benzie and Strain (1996) [45]. Briefly, 100 µL of both EPP and NEPP extracts were mixed with 900 µL of the FRAP reagent. Absorbance was measured at 593 nm in a UV–visible spectrophotometer (Evolution 300, Thermo-Scientific, Oxford, UK) precisely 4 min from the commencement of the reaction. The FRAP reagent composition comprises 0.3 M of acetate buffer, a 10 mM 2,4,6-tripyridyl-s-triazine (TPTZ) solution in a 40 mM HCl solution, and FeCl_3_·6H_2_O solution in the following proportions: 20 mL of acetate buffer, 2 mL of TPZP, and 2 mL of FeCl_3_·6H_2_O. Trolox was used as the standard, and the results were expressed as mg Trolox equivalents (TE)/g of the sample in d.w.

### 3.8. Statistical Analysis

The data were processed using R studio, version 4.0.5 (R Foundation for Statistical Computing, Vienna, Austria). All assays were conducted in triplicate. Normality was determined through the Shapiro–Wilk test. The homogeneity of variances was analysed using the Bartlett test. The *t*-student test was conducted to determine significant differences at *p*-value < 0.05. Correlation analysis were performed using the Pearson correlation test for the relationships between the functional properties and fibre composition parameters.

## Figures and Tables

**Table 1:** Percentages (%) of neutral sugars, uronic acids, cellulose, hemicellulose, and pectin in lemon peel (LP) and lemon peel hydrolysed by pectinesterase (LPp) ^§^.

Composition (%)	LP	LPp
Rhamnose	3.3 ± 0.2	24.1 ± 1.2 *
Fucose	1.1 ± 0.3 *	0.2 ± 0.2
Arabinose	31.6 ± 1.4 *	9.3 ± 0.9
Xylose	9.4 ± 1.3 *	0.8 ± 0.2
Mannose	7.3 ± 0.3 *	4.4 ± 0.3
Galactose	21.4 ± 0.6	25.5 ± 0.8 *
Glucose	9.1 ± 0.2	20.8 ± 1.2 *
Uronic acids	16.9 ± 0.1 *	14.9 ± 0.1
Cellulose ^1^	8.2 ± 0.2	18.7 ± 1.0 *
Hemicellulose ^2^	18.7 ± 1.2	7.5 ± 0.4 *
Pectin ^3^	73.1 ± 1.1	73.8 ± 0.7

^§^ Values are expressed as mean ± SD (*n* = 3). * Indicates significant differences (*p* < 0.05) among the samples. ^1^ Cellulose: glucose × 0.9; ^2^ hemicellulose: (fucose + xylose + mannose + (glucose × 0.1)); ^3^ pectin: (rhamnose + arabinose + galactose + uronic acids).

**Table 2:** Sugar ratios for characterisation of pectin and hemicellulose from lemon peel (LP) and lemon peel hydrolysed by pectinesterase (LPp) ^§^.

Parameter	LP	LPp
Mannans to hemicelluloses contr ^1^	0.8 ± 0.1	5.7 ± 1.6 *
Linearity of pectin ^2^	0.3 ± 0.0	0.3 ± 0.0
Rhamnose and uronic acid contr ^3^	0.2 ± 0.0	1.6 ± 0.1 *
RG-I Branching ^4^	16.1 ± 1.3 *	1.5 ± 0.1

^§^ Values are expressed as mean ± SD (*n* = 3). * Indicate significant differences (*p* < 0.05) among the samples. Lemon peel (LP); lemon peel hydrolysed by pectinesterase (LPp). ^1^ Contribution of mannans to hemicelluloses: mannose/xylose; ^2^ linearity of pectin: uronic cids/(fucose + rhamnose + arabinose + galactose + xylose); ^3^ contribution of rhamnose and uronic acids to pectins: rhamnose/uronic acids; ^4^ branching of RG-I: (arabinose + galactose)/rhamnose.

**Table 3:** Functional properties of lemon peel (LP) and lemon peel treated with pectinesterase (LPp).

Functional Property	LP	LPp
SWC (mL water/g)	9.9 ± 0.07 *^,1^	2.6 ± 0.0
WRC (g water/g)	1.1 ± 0.0 *	0.8 ± 0.1
FAC (g oil/g)	9.3 ± 0.6 *	2.7 ± 0.2

^1^ Values are expressed as mean ± SD (*n* = 3). * Indicate significant differences (*p* < 0.05) among the samples. Lemon peel (LP); lemon peel treated with pectinesterase (LPp). SWC (swelling capacity); WRC (water retention capacity); FAC (fat absorption capacity).

## Data Availability

Data are contained within the article and Supplementary Materials.

## Supplementary Materials

The following supporting information can be downloaded at: https://www.mdpi.com/article/10.3390/molecules29010269/s1, Figure S1: Chromatograms of (a) standard mix I for LP sample; (b) standard mix II for LPp sample; (c) LP sample; (d) LPp sample. Different peaks are 1 (rhamnose); 2 (fucose); 3 (arabinose); 4 (xylose); 5 (allose); 6 (mannose); 7 (galactose); 8 (glucose).
