# Supplementary material for: Chemical Composition, Functional and Antioxidant Properties of Dietary Fibre Extracted from Lemon Peel after Enzymatic Treatment
Source: Molecules. 2024 Jan 4;29(1):269. doi: 10.3390/molecules29010269 (PMC10780729; doi:10.3390/molecules29010269)
Supplement: Supplementary file 1 [file molecules-29-00269-s001.zip › molecules-2748426-supplementary.pdf]

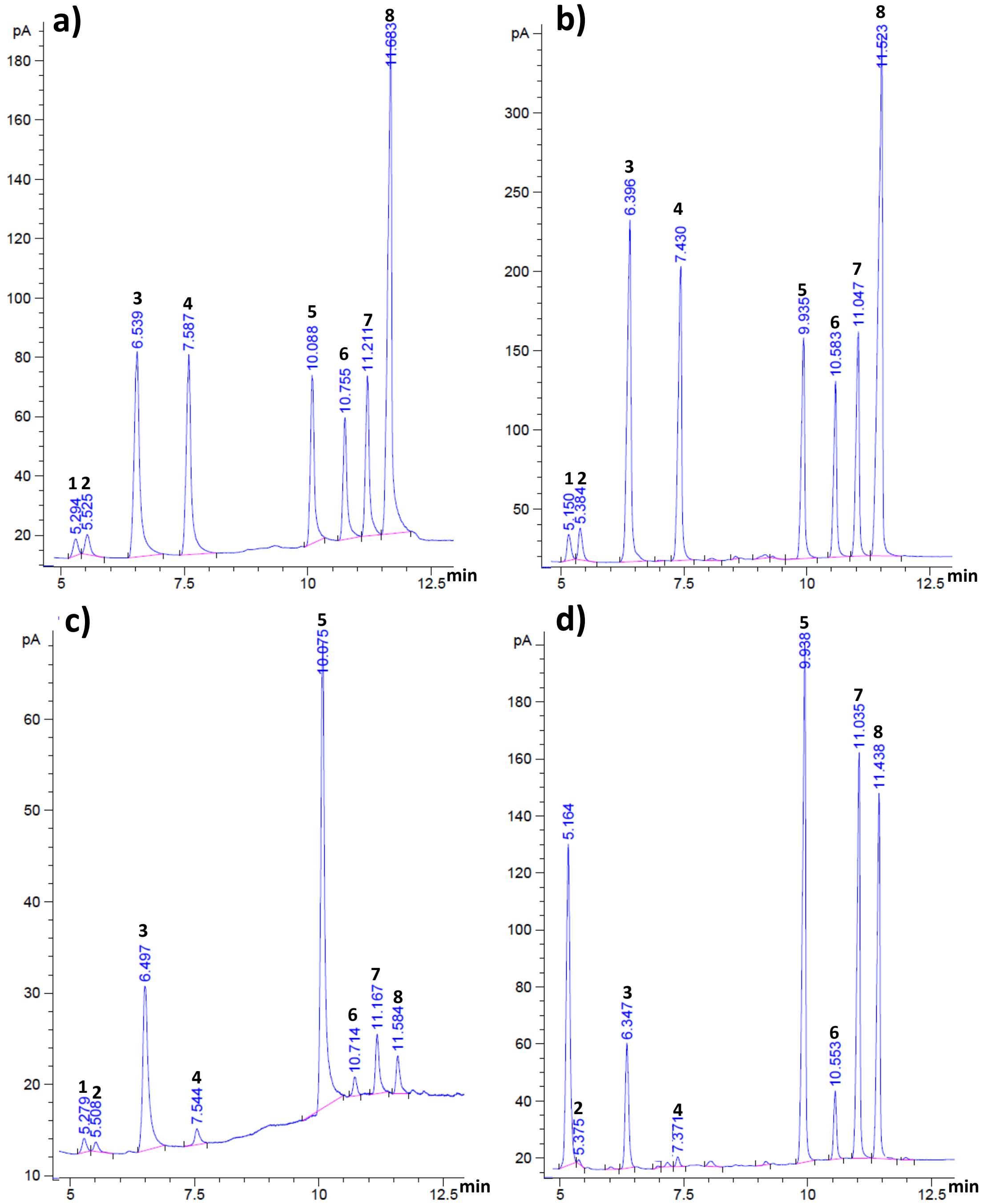

Figure S1. Chromatograms of a) standard mix I for LP sample; b) standard mix II for LPp sample; c) LP sample; d) LPp sample. Different peaks are 1 (rhamnose); 2 (fucose); 3 (arabinose); 4 (xylose); 5 (allose); 6 (mannose); 7 (galactose); 8 (glucose).
